# Exosomes for Diagnosis and Therapy in Gastrointestinal Cancers

**DOI:** 10.3390/ijms21010367

**Published:** 2020-01-06

**Authors:** Maria Principia Scavo, Nicoletta Depalo, Valeria Tutino, Valentina De Nunzio, Chiara Ingrosso, Federica Rizzi, Maria Notarnicola, Maria Lucia Curri, Gianluigi Giannelli

**Affiliations:** 1 Personalized Medicine Laboratory, National Institute of Gastroenterology “S. De Bellis” Research Hospital, Via Turi 27, Castellana Grotte, 70013 Bari, Italy; gianluigi.giannelli@irccsdebellis.it; 2 Institute for Chemical-Physical Processes (IPCF)-CNR SS Bari, Via Orabona 4, 70125 Bari, Italy; n.depalo@ba.ipcf.cnr.it (N.D.); c.ingrosso@ba.ipcf.cnr.it (C.I.); f.rizzi30@studenti.uniba.it (F.R.); marialucia.curri@uniba.it (M.L.C.); 3 Laboratory of Nutritional Biochemistry, National Institute of Gastroenterology, “S. de Bellis” Research Hospital, Via Turi 27, Castellana Grotte, 70013 Bari, Italy; valeria.tutino@irccsdebellis.it (V.T.); valentina.denunzio@irccsdebellis.it (V.D.N.); maria.notarnicola@irccsdebellis.it (M.N.); 4 Dipartimento di Chimica, Università degli Studi di Bari Aldo Moro, Via Orabona 4, 70125 Bari, Italy; 5 Scientific Direction, National Institute of Gastroenterology “S. de Bellis”, Research Hospital, Castellana Grotte, 70013 Bari, Italy

**Keywords:** exosomes, tumorigenesis, gastrointestinal cancers, gold immunostaining, lipidomic, therapeutic drug delivery nanovehicles

## Abstract

Exosomes are membrane-bound extracellular vesicles (EVs) released by most cells, having a size ranging from 30 to 150 nm, and are involved in mechanisms of cell-cell communication in physiological and pathological tissues. Exosomes are engaged in the transport of biomolecules, such as lipids, proteins, messenger *RNAs*, and micro*RNA*, and in signal transmission through the intercellular transfer of components. In the context of proteins and nucleic acids transported from exosomes, our interest is focused on the Frizzled proteins family and related messenger *RNA*. Exosomes can regenerate stem cell phenotypes and convert them into cancer stem cells by regulating the Wnt pathway receptor family, namely Frizzled proteins. In particular, for gastrointestinal cancers, the Frizzled protein involved in those mechanisms is Frizzled-10 (FZD-10). Currently, increasing attention is being devoted to the protein and lipid composition of exosomes interior and membranes, representing profound knowledge of specific exosomes composition fundamental for their application as new delivering drug tools for cancer therapy. This review intends to cover the most recent literature on the use of exosome vesicles for early diagnosis, follow-up, and the use of these physiological nanovectors as drug delivery systems for gastrointestinal cancer therapy.

## 1. Introduction

**Figure 1:**
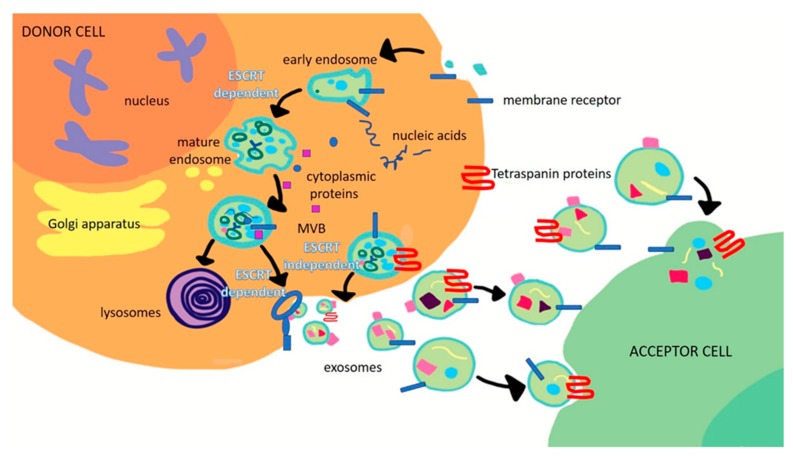
Scheme of the biogenesis of exosomes mainly occurring through two pathways, namely endosomal sorting complex required for transport (ESCRT)-dependent or -independent.

In the last decade, a significant number of studies have focused their attention on the exosome-mediated cross talking that occurs between cancer and normal cells, especially in the tumor microenvironment, which is able to promote the activation of signaling pathways and, consequently, reprogram the functions of recipient cells by means of their cargo transfer [1]. Following such a trend, several examples have been reported in the literature, proving the ability of exosomes to favor and support tumor growth and spread and metastatic dissemination in many types of cancer [2,3,4]. Exosomes are extracellular nanovesicles (EVs) characterized by a diameter in the range of 30 to 150 nm. They are produced by many types of cell and have been found in various bodily fluids, including plasma, urine, amniotic fluid, saliva, gastric acid, and malignant ascites [5]. Exosomes are natural nanocarriers for different molecular constituents and their content strictly depends on the type of cell and its physiological conditions. Different types of proteins, including heat shock proteins (HSPs); tetraspanins; integrins; and vacuolar, sorting-associated, and Rab family proteins, can be found in exosomes, thus resulting in them being specifically associated with different types of cell. Some of them are involved in exosome biogenesis processes. For example, tumor susceptibility gene 101 (TSG101), ALG-2-interacting protein X (ALIX), CD63, CD81, and HSP70 are the most representative proteins for exosomes, and consequently, are typically considered reliable housekeeping markers since they are able to unequivocally identify the exosomes with respect to other extracellular vesicles [6]. Exosomes are formed by the generation of intraluminal vesicles (ILVs) that are derived from the inward budding of the limiting membrane of multivesicular bodies (MVBs). When MVBs containing ILVs fuse with the plasma membrane, ILVs are released into the extracellular space, thus producing exosomes. This step is controlled by specific proteins that belong to the Rab family. The biogenesis process of exosomes can be endosomal sorting complex required for transport (ESCRT)-dependent. According to this pathway, four complexes of proteins (ESCRT-0, ESCRT-1, ESCRT-2, and ESCRT-3), as well as TSG101 and ALIX proteins, are involved in the transition from endosomes to exosomes [7,8]. This process requires ubiquitination of the cytosolic tail of endocytosed receptors. Tsg 101, which belongs to the ESCRT-I complex, is enrolled to form a complex with the ubiquitinated cargo proteins and activates the ESCRT-II complex, which leads to oligomerization and the formation of the ESCRT-III complex. The ESCRT-III complex is important for the requisitioning of MVB proteins and enrollment of enzymes which remove the ubiquitin tag from the cargo proteins before placing them into the ILVs. Finally, the ESCRT-III complex is degraded by an ATPase. A proteomic analysis of members of the ESCRT complex, such as Alix and Tsg101, in the exosomes, has supported ESCRT-dependent exosome biogenesis [9]. On the other hand, ubiquitination is not systematically required for the biogenesis of exosomes in different cell types; in fact, in melanocytes, sequestration of the premelanosomal protein Pmel17 in ILVs appears to be ESCRT-independent [10]. Different types of machinery for exosome biogenesis have been suggested by different studies and could exist in different cell types or, alternatively, co-exist in the same cell type, and the use depends on the proteins vehiculated [11] into the exosomes and the function of these proteins. In fact, the formation of exosomes can also occur by means of the generation of ESCRT-independent ILVs, which involves specific tetraspanins, such as CD63 and CD81, and lipids, namely ceramide and cholesterol molecules [12,13] (Figure 1). However, the different mechanisms behind exosome production that also determine their cargo, are still largely unknown, likely being dependent on the types and status of the cells. Along with proteins, exosome cargo includes *mRNA*, *DNA*, micro*RNA* (*miRNA*), and long noncoding *RNA* (*LncRNA*) that can induce genetic and epigenetic modifications in recipient cells in terms of activity and functions [13]. Moreover, lipids, such as cholesterol, phospholipids, glycerophospholipids, sphingolipids, and ceramides, are essential components of exosomes as they form a much more stable bilayer membrane structure [14]. Tumor-associated exosomes are larger than those derived from healthy cells and, interestingly [15], are enriched with different types of mediators of tumorigenesis, including oncoproteins, growth factors, immunomodulatory molecules, lipids, nucleic acids, and numerous metabolites, that arise from the cytoplasm of donor cancer cells and strongly reflect the tumor conditions [4]. Indeed, two major challenges in the oncological field are currently represented by the identification, in the tumor cell-derived exosomes, of components that induce pro-tumorigenic effects in tumorigenic and metastatic processes and understanding the pathways that allow the inclusion of these components in exosomes [16]. Several examples of exosome biomarkers have already been identified and documented in recent literature for the diagnosis and prognosis of specific types of cancer [17,18].

Indeed, different studies have proved that glypican-1 (GPC-1) represents a diagnostic exosome marker for pancreatic, breast, and colorectal cancer (CRC). Moreover, high expression levels of CD9 and CD147 have been found in exosomes derived from CRC patient serum, while a decrease in CD147 has been observed after cancer removal surgery. PSA, CD9, and gammaglutamyltransferase 1 (GGT1), a cell-surface enzyme, isolated from human serum resulted in significantly higher prostatic cancer (PC) than in benign prostatic hypertrophy (BPH) patients [18]. The dysregulation of *miRNA* and cancer progression have been demonstrated to be strictly correlated in different types of cancer, such as cervical carcinoma, malignant human breast tumors, and prostate cancer. Furthermore, hepatoma cell-derived exosomes have been reported to deliver *miR-103* into endothelial cells, thus inducing metastasis. It has also been documented that *LncRNAs* delivered by exosomes, such as *Lnc-sox2ot*, *Lnc-h19*, and *LncRNA-ARSR*, are closely associated with tumor progression [14,19,20,21].

While an extensive characterization of the protein and nucleic acid content of exosomes has been carried out, revealing the association between different exosomes and tumors and thus suggesting their potential as markers, another relevant class of bio-molecules, lipids, has been overlooked in studies investigating exosome composition, in spite of the renowned lipid implication in multiple aspects of exosome biogenesis and function.

Recent technological developments in lipidomics have allowed researchers to deeply investigate the role of lipids carried by exosomes, also from the perspective of their potential use as biomarkers for cancer detection and progress [14]. For example, Brzozowski J. S. et al. demonstrated that exosomes derived from non-tumorigenic prostatic cells are enriched with fatty acids, glycerolipids, and prenol lipids, while the content in sterol lipids, sphingolipids, and glycerophospholipids was found to be significantly higher in the exosomes isolated from cancer pancreatic cells [2]. Furthermore, Smith Z. J. et al. indicated that phospholipids conveyed by exosomes represent potentially specific biomarkers for the early detection and diagnosis of breast cancer [22].

Interestingly, tumor-derived exosomes can be used as physiological therapeutic nanovectors, since they possess multiple relevant features that make them eligible as ideal drug delivery systems. Indeed, they represent a superior choice when compared to conventional synthetic drug delivery nanosystems, since they overcome the limited biocompatibility and/or low tolerance [23]. Drug-loaded exosomes are able to preserve the functionality of therapeutic compounds from the degradation of the extracellular environment and, at same time, they possess a very high degree of biocompatibility, a minimal immunogenicity effect, and a surface enriched with specific proteins, such as integrins, which are useful for the active targeting of cancer sites [24]. In different types of cancers, the ability of exosomes to deliver drugs inside the parental cancer cells by endocytosis has been proved, thus ensuring an increased cytotoxicity in the recipient cancer cells [25].

In this review, we intend to give an overview of how exosome-mediated cell-cell communication can regulate carcinogenesis in gastro enteric cancers and how exosomes represent an original and effective diagnostic tool for specific gastrointestinal cancers thanks to the peculiar biological characteristics of their membrane surface. Original preliminary results on different lipid profiles in the membranes of exosomes isolated from human colon epithelial cells and malignant human colon adenocarcinoma are also presented. Finally, the potential of exosomes as drug delivery nanocarriers for the treatment of gastrointestinal cancers will also be discussed, envisioning a future effective therapeutic alternative with respect to conventional disease management.

## 2. Role of Exosomes in Tumorigenesis of Gastrointestinal Cancers

During carcinogenesis, microenvironment modification and cell transformation take place to transform normal tissue into primary cancer tissue or metastatic sites. Different studies have referred to the involvement of exosomes in cancer development and their role in metastatic spread, as well as gastrointestinal and most generically gastroenteric tumorigenesis. Exosomes containing mutated *p53 DNA* were isolated from the serum of patients affected by pancreatic cancer and their detection was ascribed to the malignance of cancer [26]. Costa Silva B. et al. proved, in a preclinical in vivo study, that pancreatic ductal adenocarcinoma (PDAC)-derived exosomes are involved in liver pre-metastatic niche formation and consequently support the spread of liver metastasis. The authors found that the macrophage migration inhibitory factor (MIF) is highly expressed in PDAC-derived exosomes, extracted from stage I PDAC patients, and that liver pre-metastatic niche formation and metastasis can be prevented by an MIF blockade. These results indicated that MIF delivered by PDAC-derived exosomes may represent a valid prognostic marker for the development of PDAC liver metastasis [27]. Herrera M. et al. performed an investigation based on differential representation and enrichment analyses based on non-coding *RNAs* (*ncRNAs*) that highlighted the presence of several prominent differences between the *ncRNA* expression levels in exosomes isolated from normal and cancer-associated fibroblasts (CAF) in CRC. Their findings suggested that *ncRNAs* delivered by exosomes are potential biomarkers for CRC and that CAF-derived exosomes are mediators of specific cross-talk between CAFs and colon cancer cells [28]. Similarly, in the presence of CRC, it has been reported that *miR-10b*-containing exosomes derived from cells in the microenvironment of the tumor, such as CAFs, can contribute to proliferation and promote disease progression by modulating surrounding stromal cells. The *miR-10b*-containing exosomes have been demonstrated to be able to reduce fibroblast proliferation and promote the expression of transforming growth factor-beta (TGF-β) and smooth muscle (SM) α-actin, inducing the activation of normal fibroblasts to CAFs, with their consequent expression of myofibroblast markers. These CAFs have been demonstrated to promote in vitro and in vivo CRC growth [29]. 30. Ju Q. et al. proved that *miR-21-3p* and *miR-769-3p* delivering exosome activation, whose secretion was enhanced by p53 R273H mutation, were able to induce the activation of fibroblasts in the tumor microenvironment and lung tissues. They proved that this process supports the formation of premetastatic niches to promote the pulmonary metastasis of CRC cells [30]. A study reported by Cooks T. et al. revealed that *miR-1246*-enriched exosomes were selectively secreted by CRC cells harboring gain-of-function (GOF) mutant p53. *miR-1246*, as unique cargo of mutp53-derived exosomes potentially amenable for therapeutic and diagnostic applications in CRC cancer, was identified. They found that the uptake of mutant p53-derived exosomes triggered the formation of a distinct sub-population of mutant p53-reprogammed tumor-associated macrophages involved in cancer progression and metastasis [31].

Specific circular *RNAs *(*circRNAs*), which are covalently closed single-stranded RNA molecules derived from exons by alternative *mRNA* splicing, were found to be enriched and stable in exosomes isolated from the human serum of patients affected by colorectal cancer or pancreatic cancer compared with those in exosomes from healthy controls, thus highlighting the implication of exosomal *circRNAs* in the pathogenesis and/or progression of cancer [32,33,34]. Zhou J. et al., demonstrated that *miRNA-21*-containing exosomes derived from hepatocellular carcinoma (HCC) cells are characterized by a significant ability to convert normal hepatic stellate cells (HSCs) to CAFs. Furthermore, the *miRNA* contained in CAF-derived exosomes, which is involved in hepatocellular carcinoma (HCC) development, has been demonstrated to enable the conversion of normal HSCs, hepatic stellate cells, into CAFs, by directly targeting the phosphatase and tensin homologue (PTEN), with a consequent secretion of different growth factors, such as vascular endothelial growth factor (VEGF), fibroblast growth factor-2 (FGF-2), and growth factor-beta (TGF-β) [35].

Recently, Yang N. et al. found that *TGF-β* is highly expressed in HCC-derived exosomes and the authors studied the role of neutrophil infiltration in HCC tissues by means of Axl-induced CXCL5, with a consequent promotion of tumor progression in HCC models [36,37]. Other reports have elucidated the role of exosomes during the process of metastasis in pancreatic cancer. Gutkin A. et al. proved that the transformation of non-malignant fibroblasts into telomerase-positive cells is mediated by the transcription of enzyme telomerase (*hTERT mRNA*) that is delivered by exosomes, thus suggesting their role in modifying the cancer microenvironment [38]. In gastric cancer (GC), *miR-21*-regulated PTEN/PI3K/AKT signal transduction has been detected in exosomes and the involvement of these micronucleic acids in apoptosis inhibition and in cisplatin resistance was shown to increase [39]. Another bioactive molecule detected in GC-derived exosomes, *ZFAS1*, responsible for MAPK signal regulation and EMT transcription factors, has been found to be involved in cell cycle progression, inducing an enhancement of cancer growth and metastasis [40]. Moreover, exosomes can regenerate stem cell phenotypes and convert them into cancer stem cells by regulating the Wnt pathway receptor through the regulation of stem-related signaling pathways [41]. In a very recent study, we investigated the specific role in carcinogenesis of both FZD10, a protein directly involved in carcinogenesis and tumor proliferation, and its messenger *RNA* (*FZD10-mRNA*), which have been demonstrated to be carried by exosomes isolated from different gastrointestinal cancer cells, namely human colorectal adenocarcinoma cells (CaCo-2), metastatic SW-620 colon cancer cells, hepatocellular carcinoma cell lines (Hep-3B, HLF, and HLE cells), liver hepatoma cells (PLC-5), gastric carcinoma cells (derived from metastatic site, N-87), human gastric carcinoma cells (derived from metastatic site, HGC-27), and human intrahepatic cholangiocellular carcinoma cells (HUCCT-1). In particular, for each cell line, the restoration of viability in the *FZD10-mRNA*-silenced cells, which drastically decreases their viability, has been proved in vitro by cell incubation with the corresponding FZD10- and *FZD10-mRNA*-delivering exosomes isolated from the non-silenced counterpart. The in vitro findings suggest that FZD10- and *FZD10-mRNA*-delivering exosomes may be potential messengers of cellular transformation that are able to play an active function in the long-distance metastatization process [42]. The involvement of FZD10 vehiculated in small extracellular vesicles (sEVs) in the control of cancer progression and cancer cell modification has also been suggested by another previous clinical study [43]. In particular, the expression of FZD10 in EVs extracted from the plasma of patients affected by CRC and GC has been investigated, before and after treatment (surgery, chemotherapy, and metastasis removal), by using the protein expression in the EVs from the plasma of healthy subjects as a reference. The results have highlighted that the level of protein expression in oncological patients, in each investigated cell line, was higher than that observed for healthy donors, thus providing a significant indication of the pathological condition. Interestingly, comparable average values of the FZD10 level in EVs from patients at the end of treatment and in healthy subjects have been found.

**Figure 2:**
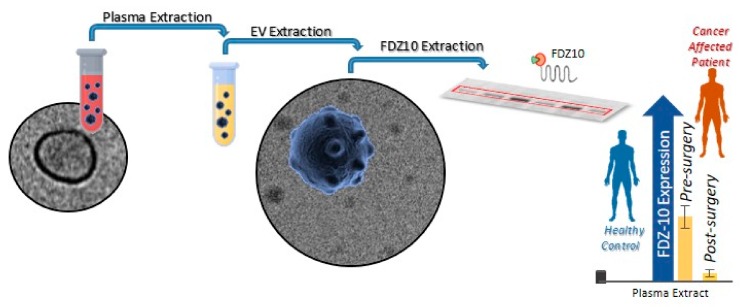
Schematic illustration of the possible use of the FZD10 vehiculated in human plasma extracellular vesicles (EVs) as a prognostic biomarker for the early diagnosis of two specific gastrointestinal cancers (colorectal cancer (CRC) and gastric cancer (GC)) and for monitoring the treatment response by an evaluation of the protein expression level in healthy control subjects vs. oncological patients.

A clinical study has clearly proved that a reliable potential biomarker with a valuable prognostic role for the early diagnosis of two specific gastrointestinal cancers, namely CRC and GC, and for monitoring the treatment response, can be effectively represented by FZD10 contained in the plasma EVs by an evaluation of its expression level (Figure 2).

## 3. Detection of Exosome Proteins as Biomarkers for Gastrointestinal Cancers

Preliminary steps for the detection of exosome proteins as biomarkers are represented by exosome extraction from biological fluids and characterization in terms of their size, morphology, and protein cargo, to verify the effectiveness of the isolation technique and univocally identify the isolated exosomes. Several methods have been exploited for exosome isolation: ultracentrifugation, filtration, commercial kits, microfluidic devices and polymer-based precipitation, chromatography, and immunoaffinity isolation [44]. Among them, chromatography size exclusion ensures the extraction of exosomes with a good grade of purity. Compared to centrifugation protocols, which may require multiple ultracentrifugation steps, also at a high speed and for long time, the chromatographic method may better preserve the integrity of exosomes that the centrifugal force compromises. However, chromatography size exclusion requires a long running time and provides a diluted suspension of exosomes; furthermore, it is very expensive [45,46]. In a recent paper, Kumar G. et al. investigated the methods for exosome extraction from a cell supernatant by comparing ultracentrifugation with four different commercial kits [47]. The results highlighted that ultracentrifugation is the most suitable method for the extraction of vesicles from cell supernatant, in terms of the purity, quantity, and integrity, provided that it is properly applied; use of the ultracentrifugation method should consist of multiple centrifugation cycles carried out for a limited time and at a specific speed. Indeed, such a method allows contamination derived from the microvesicles to be eliminated, and a reasonable quantity of exosomes to be obtained for the following steps of diagnosis. Currently, ultracentrifugation is generally considered the gold standard in exosome extraction and it is widely used in laboratories [48], though the most suitable technique for exosome isolation ultimately depends on the type of biological fluid, the aim of the investigation, and the availability of equipment and resources [49].

**Figure 3:**
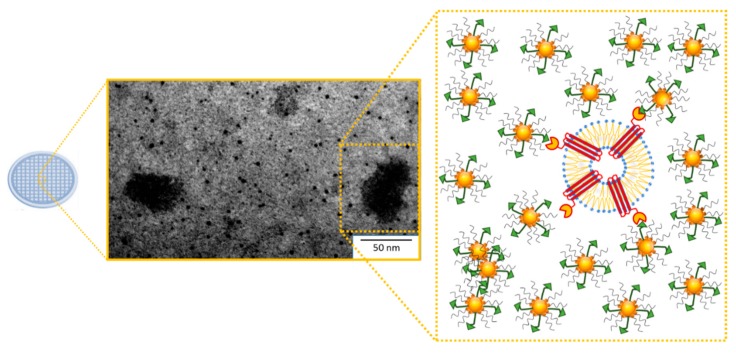
Immunogold labeling for the FZD10 protein on the exosome surface. Representative TEM micrograph obtained with the positive staining of exosomes derived from gastrointestinal cancer cells after their linking to FZD10 antibody-functionalized Au NPs by molecular recognition, along with the corresponding schematic representation.

After extraction, exosomes can be conveniently processed for physical characterization by means of Dynamic Light Scattering (DLS), Transmission Electron Microscopy (TEM), and Scanning Electron Microscopy (SEM) [50]. In the typical experimental procedure, the obtained exosome suspension, after isolation and physical characterization, may be homogenized. Then, protein quantification and characterization are performed using classic methodologies for proteins assays, e.g., electrophoresis and immunoblotting detection with specific antibodies, the radiolabeling of proteins, Edman degradation, RP-HPLC, direct or indirect ELISA, and spectroscopic measurement. These methods allow the identification and quantification of specific contents of proteins from the total extract of exosomes. Actually, different types of kit are able to detect the total exosomes *RNA*, internal proteins, and membrane compounds, by using generic fluorescent labeling. Dean I. et al. used a generic labeled probe for the membrane of exosomes to detect the exosomes in cell cultures [51]. Moreover, the possibility of localizing exosome proteins using immunogold labeling detection by TEM or other types of microscopies, including confocal and optical microscopy, offers an interesting alternative. TEM is the most common type of electron microscopy for exosome visualization and morphological characterization by imaging [52]. Recently, a TEM grid was used as a support for depositing Au NPs (gold nanoparticles) that were surface functionalized with FZD10 protein primary antibody in order to possibly detect, at the surface of the exosomes, the presence of the protein that represents the gastrointestinal cancer-associated marker [49]. In Figure 3, representative TEM micrographs of exosomes isolated from gastrointestinal cancer cells and bound to FZD10 antibody-functionalized Au NPs have been reported. The obtained data proved that molecular antigen/antibody recognition only occurs when Au NPs are functionalized with FZD10 antibody, thus suggesting the presence of FZD10 on the membrane of exosomes [39] and, ultimately, indicating its involvement in gastric and colon carcinogenesis.

Additionally, atomic force microscopy (AFM) has been used to image exosomes by using a silicon or silicon nitride probe [53]. In air-mode AFM, sample preparation for exosome imaging only requires immobilized exosomes on freshly cleaved mica and demonstrates that hydrated exosomes retain their shape and size, also enabling cell derivation. By using such a technique, Yuana et al., after the isolation of exosomes from the blood of healthy donors and oncological patients, imaged them upon their immobilization on a modified mica surface coated with an antibody against CD41. Several studies have reported on the characterization of CD41-positive exosomes using flow cytometry. For example, Yuana Y. et al. proposed an alternative method suitable for a sensitive and reproducible detection of antigen-positive exosomes, and found that their plasma concentration was 1000-fold higher than that measured by conventional flow cytometry. This confirms that it is possible to coat with other antibodies against the proteins of exosomes with specific antigens, as well as to image the resulting complexes [53].

## 4. Alteration of the Exosomal Lipid Profile in Gastrointestinal Tumors

**Figure 4:**
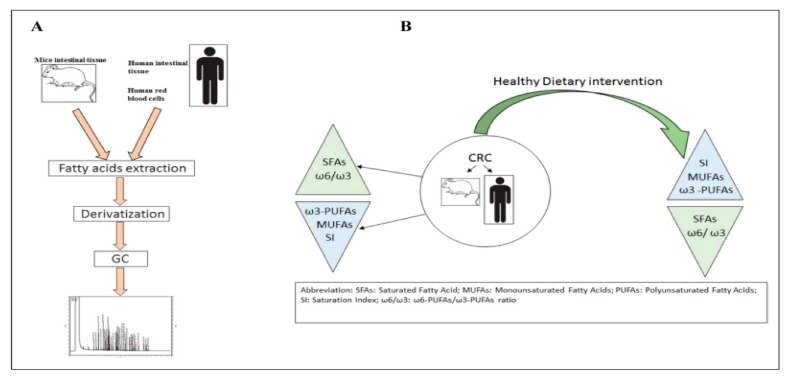
Panel (**A**): Main steps of lipidomic analysis; Panel (**B**): Dietary intervention effects (reproduced with permission from [54]).

Several studies have shown that an alteration of the cellular lipidomic profile takes place in the presence of different types of tumor, such as CRC (Figure 4) [54,55].

**Figure 5:**
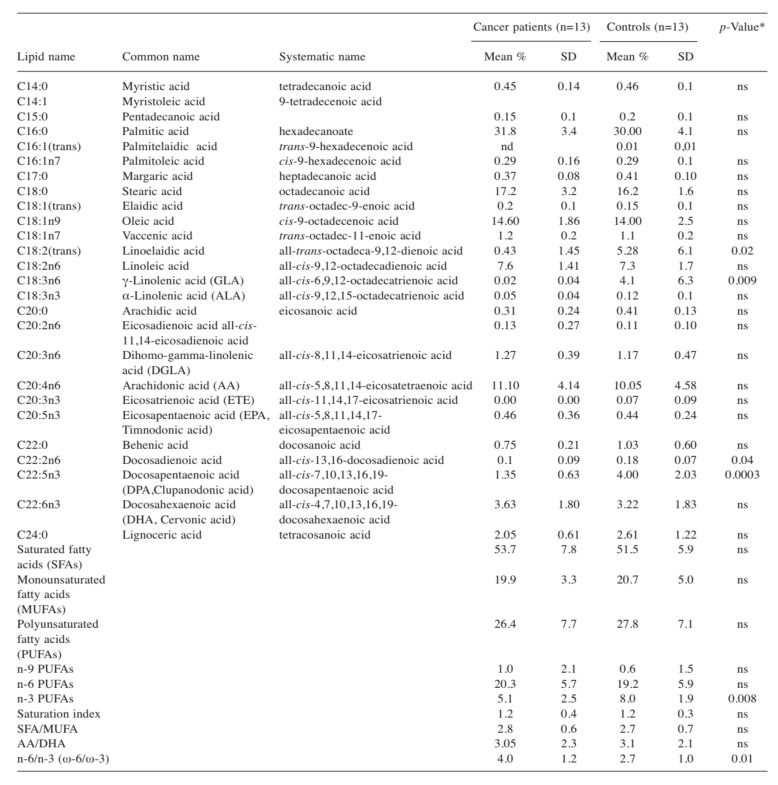
Mean percentage of the fatty acid composition of red blood cell membranes in cancer patients and controls (reproduced with permission from [61]). Mann-Whitney test, differences were considered significant at *p* < 0.05; ns: no significant; nd: no detected.

Gastrointestinal cancer, like other types of cancers, is a multifactorial disease [56] provoked by genetics, familiar, and environmental factors. It is now well-established that the metabolism of lipids and phospholipids plays a crucial role during colorectal carcinogenesis, tumor invasion, and metastasis [57,58,59]. In colon cancer tissues, the level of lysophosphatidylserine and lysophosphatidylserine, measured by liquid chromatography-tandem mass spectrometry (LC–MS/MS), has been found to be significantly higher than that in surrounding normal tissues, thus suggesting that the lysophospholipids may play an important role in the development of CRC [60]. Previously, the lipidomic profile in red blood cell membranes of patients with CRC has been demonstrated to be altered when compared to control subjects. In particular, in cancer patients, a high value of the omega-6/omega-3 ratio has been detected, thus confirming the contribution of inflammation in cancer development (Figure 5) [61]. Proinflammatory stimuli, such as eicosanoids synthesized by both omega-3 and omega-6 polyunsaturated fatty acids (PUFAs), cause a microenvironment suitable for cancer progression, invasion, and metastasis [62]. The lipidomic approach used to assess tissue inflammation is based on the determination of changes in the membrane lipid structure and the use of new proinflammatory biomarkers involved in cancer and its metastatic process.

In this respect, studies conducted on tumor tissue of metastatic CRC patients have shown the presence of low levels of eicosapentaenoic acid (EPA) and high γ-linolenic acid (GLA) levels compared to patients without metastases [63]. Furthermore, high values of the arachidonic acid/eicosapentaenoic acid (AA/EPA) ratio, which is an excellent index for evaluating inflammation, have been detected in the tumor tissues from CRC patients with metastases [64]. Indeed, during inflammation, the eicosanoids derived from fatty acids and involved in lipid signaling molecules play an important role in degeneration of the disease. Wang et al., by using an LC-MS/MS-based lipidomic approach, found that obesity induces inflammation in colon tissues with a consequent increase in the expression of soluble epoxide hydrolase (sEH) and its metabolite eicosanoids, namely, fatty acid diols [65]. The lipidomic profile reveals soluble sEH as a therapeutic target of obesity-induced colon inflammation. The new approach based on discriminatory analyzes of the phospholipid profile and lysophospholipid ratio is used for the evaluation and diagnosis of tumors, in particular, to discriminate a tumor tissue from a normal one or colon epithelial cells isolated from the tumor compared to non-tumor samples of colon cancer patients [66]. The lipidomic assay allows an evaluation of all fatty acids and the composition of biological membranes, as well as an investigation of their involvement in the migration and remodeling phenomena. Exosome membranes are formed of a variety of metabolites and components of the lipid cell membrane. For the first time, in 2013, in vitro work demonstrated the lipid species composition of exosomes and compared it with the parent prostate cancer cell line PC-3, showing that the exosomes lipid composition retains the same structural order of the cell membranes that they are derived from.

The high glycosphingolipid content in exosomes is likely responsible for the stability of their membranes in the intracellular environment and is essential for their role in cell-cell communication [11]. In 2016, Haraszti R. A. et al. established that there is a clear difference between the composition of source cells and exosomes, which indicates that lipids and proteins play different roles as a function of their localization, whether in cells or in exosomes [67]. Although the enrichment in commonly used exosome markers has been defined as source-cell-type dependent, all exosome markers can be found in microvesicles as well [67]. More recently, in exosomes isolated from the serum of patients with pancreatic cancer, dysregulation of the lipid composition was detected. Therefore, such a feature has been exploited for the identification and use of biomarkers for the diagnosis or pathological relationship with the progression of pancreatic cancer [68]. Preliminary experiments that we have recently carried out have demonstrated the presence of a different lipidomic profile in the membranes of exosomes derived from human colon epithelial cells (HCEC-1CT) and human colon adenocarcinoma Caco-2 cells. After exosome isolation, according to the Folch method, the total lipids have been extract [69]. The fatty acid methyl esters (FAME) have been obtained by adding toluene and boron trifluoride-methanol solution and the upper phase has been collected and injected into a gas chromatograph. The separation of FAME has been carried out on a gas chromatograph capillary column (SGE Europe Ltd., Milton Keynes, UK), as previously reported [63,67]. The quantification of FAME has been performed using a mixture of standards (Supelco 37-Component FAME Mix, Sigma-Aldrich, Milan, Italy). Table 1 shows the mean percentage of the fatty acid composition of exosome membranes extracted from HCEC-1CT and Caco-2 cell lines. Compared to exosomes extracted from human colon epithelial cells, those isolated from Caco-2 cells showed an altered lipidomic profile characterized by high levels of PUFAs (3.06% vs. 2.73%). Of these PUFAs, the largest contribution is given by omega-6 fatty acids (2.52% vs. 2.25%). In particular, in the exosome membranes extracted from Caco-2 cells, high levels of linoleic acid (LA), γ-linolenic acid (GLA), and arachidonic acid (AA) were determined. Moreover, lower levels of *omega-3* fatty acids, such as eicosapentaenoic acid (EPA) and α-linolenic acid (ALA), have been found in exosomes extracted from Caco2 cells than in those derived from HCEC-1CT. In exosomes extracted from Caco2 cells, high levels of the n-6/n-3 and AA/EPA ratio have also been determined, confirming the proinflammatory contribution of omega-6, and in particular, of AA, in CRC tumorigenesis (Table 1).

## 5. Cancer-Derived Exosomes as Drug Delivery Vehicles for the Treatment of Gastrointestinal Cancers

Comprehensive knowledge of the lipid and protein composition of exosomes is essential for using them as vehicles, and creating alternative and personalized drug-based nanovectors for cancer therapies. The small size of these vesicles allows passive transport in the cancer sites by exploiting the enhanced permeability and retention effect (EPR) [70]. The use of exosomes that are natural nanostructures may be a relevant example of theranostic nanomedicine [71]. Indeed, exosomes extracted from serum or plasma have been studied in cancer nanomedicine in different fields, such as nanoanalytical contrast reagents, drug delivery, and nanoformulation [72]. Exosomes, as ideal drug delivery vehicles with a low immunogenicity, high biocompatibility, and high efficiency, have been effectively used in breast cancer triple negative (TNBC) therapy. Indeed, a formulation of exosome loaded with erastine has been proved to be able to target TNBC cells with an overexpression of folate receptor, which induced the death of ferroptosis, an iron-dependent lipid peroxide cell, caused by inhibition of the cystine/glutamate transporter with ROS (Reactive Oxygen Species) overgeneration [73]. Another prominent advantage of exosomes as vehicles for drug delivery is the reduction of cytotoxicity. In the treatment of breast and ovarian cancers, Hadla M. et al. demonstrated, in mice, the crucial role of the use of doxorubicin delivered by exosomes to reduce heart toxicity, with the retention of chemotherapy effects in the treatment cycle [74].

Although studies on the use of exosome-based drug delivery vectors in the therapy of gastroenteric cancer (i.e., colon, gastric, pancreatic, liver, and cholangio cancer disease) are still limited, few in vitro and in vivo studies have been reported, highlighting the great progress that has being made. Pancreatic cancer is one of the most aggressive gastroenteric cancers; Pascucci L. et al. proposed the use of mesenchymal stromal cells (MSCs) and their exosomes to deliver Paclitaxel (PAC), an anticancer agent, taking advantage of their ability to home in on the tumor microenvironment. Indeed, by comparing the result with the free drug, they found that MSCs are able to take up and release the drug through exosome production, with a consequent increase in anti-proliferative activity in pancreatic adenocarcinoma [75]. An interesting study has reported on the milk-derived exosomes for the oral delivery of hydrophobic chemotherapeutic drug PAC, typically used in the treatment of early-stage and advanced ovarian, breast, lung, pancreatic, and other cancers. The report evaluates the antitumor efficacy of the milk-derived exosomes loaded with PAC (ExoPAC) by using athymic nude mice treated for a xenograft transplant of lung cancer and compares it with the antitumor effect of free PAC. No significant toxicity due to Exo or ExoPAC was recorded compared to PAC alone. Moreover, a comparison of the effects due to the administration of drug-free exosome suspension with those of a low dose of ExoPAC, resulted in a small but detectable increase in the inhibition of tumors (37% of reduction). Furthermore, the higher tested dose of ExoPAC (4 mg/kg b. wt.) supplied by oral administration resulted in significant tumor inhibition (reduction of nearly 60% compared to drug-free exosome vehicles) [76]. Other studies have confirmed a relationship between cancer-derived exosomes and the modulation of immune responses in pancreatic cancer. The use of these vesicles as delivery systems for anti-cancer drugs and other molecules, such as *RNAi* against mutant kirsten rat sarcoma viral oncogene homolog (KRAS), has been demonstrated to specifically target pancreatic cancer cells. These exosomes could be isolated from a patient’s biological fluids; bioengineered and transformed to deliver anti-cancer drugs, targeting ligands; and then administered in the form of exosome-based anti-cancer vaccines [77]. In addition, a recent in vivo study has shown that the combination of canonical chemotherapy drugs used for pancreatic cancer treatment (i.e., sunitinib, gemcitabine, and all-trans retinoic acid (ATRA)) with vaccines containing dendritic cells enriched with pancreatic cancer-derived exosomes, significantly inhibits the spread of metastases and extends mice survival thanks to the presence of a higher number of activated T cells in the tumor [78]. Barok M. et al. demonstrated, by an in vitro study, that trastuzumab emtansine (T-DM1), an antibody-drug conjugate designed for the specific delivery of cytotoxic drug DM1, a derivative of maytansine, to human epidermal growth factor receptor-2 (HER2)-positive cancer cells, is able to bind to exosomes derived from HER2-positive breast cancer cells and GC cells. Furthermore, exosomes linked to T-DM1 can deliver the drug to other cancer cells, possibly inducing growth inhibition and apoptotic death of the recipient HER2-positive cancer cells. Accordingly, a new exosome-mediated mechanism of action for T-DM1 that might result in an enhanced therapeutic efficacy of T-DM1 has been pointed out [79]. Zheng P. et al. investigated the effect of tumor-associated macrophage (TAMs)-derived exosomes on the migration of GC. The gastric TAMs are primarily M2-type macrophages, namely, a subpopulation with macrophages exhibiting pro-tumorigenic activity, and the M2 exosomes promoted the in vitro and in vivo migration of GC. Interestingly, the study proved the crucial role of the apolipoprotein E (ApoE), an M2-specific and highly rich protein derived from M2 exosomes, in determining the migration potential of GC cells [80]. Wang X. et al. reported an in vitro investigation performed on HEK293T cells, which proves that exosomes represent efficient nanovectors for the delivery of *anti-miR-214*, and are thus able to reverse chemoresistance to cisplatin in GC [81]. An in vitro and in vivo study demonstrated that, after HEK293T cell transfection with hepatocyte growth factor (HGF) *siRNA*, the cell secreted exosomes containing HGF *siRNAs* that inhibited the proliferation and migration of cancer cells in GC [82]. In this context, oncological research on CRC has focused on exosome scaffold proteins derived from colon cancer and characterized by membrane-scaffold-based and protein-scaffold-based ferritin nanocages, both harboring SIRPα (signal regulatory protein α), an antagonist of CD47 on tumor cells. An evaluation of the efficacy of these delivery systems defined for protein therapeutics was conducted in terms of their ability to enhance the phagocytosis of tumor cells, by bone-marrow-derived macrophages and the consequent inhibition of in vivo tumor growth. These analyses confirm that the therapeutic index of the exosome-mediated CD47 blockade against tumor growth inhibition is higher than that of the same dose of ferritin-SIRPα. Moreover, the obtained results have highlighted the relevance of unique exosome membrane scaffolds and their protein fraction that mimics the membrane proteins in their native conformations. An enhancement of therapeutic protein delivery has also been found compared with protein-scaffold-based nanocages [83]. The exosomal membrane proteins have demonstrated a significant targeting ability, increased expression levels, enhanced solubility, and antigen immunogenicity for therapeutic purposes [6]. Very recently, Li Y. et al. documented the ability of exosomes coated with high-density antibodies to target specific ligands in CRC by using the doxorubicine (Dox) as cargo. Briefly, exosomes were isolated from A33-positive LIM1215 cell lines and loaded with Dox; then, the surface-carboxylated superparamagnetic iron oxide nanoparticles (SPIONs) were coated with A33 antibody to bind A33-positive exosomes and targeted A33-positive colon cancer cells. The results of this experiment indicated a significant tumor-targeting ability of the A33Ab-superparamagnetic NPs-Exo/Dox, which have been proven to inhibit tumor growth [84]. Exosomes have shown remarkable difference with respect to commonly used synthetic drug delivery nanosystems, such as liposomes, micelles, or other polymeric NPs, due to their natural stability and intrinsic homing properties that are determined by the composition and cell origin of the exosomes themselves [24].

## 6. Conclusions

A detailed overview of the role that tumor-derived exosomes play in cell-cell communication, to affect the tumor environment and to support the evolution of gastrointestinal cancers, as well as of the potential of exosomes as biomarkers for the diagnosis and monitoring of the therapeutic response of specific gastrointestinal cancers, has been reported and discussed. Actually, the concept of using the exosomes as biomarkers for early diagnosis is rapidly spreading, concomitantly resulting in a prominent interest in the isolation, purification, and characterization of exosomes, accomplished by different methods developed for protein and lipid detection. However, while these techniques are effective for investigating the exosome structure, they still appear insufficient for achieving a satisfactory localization of specific exosome proteins. Furthermore, a comprehensive investigation of lipid exosomes’ composition is needed to achieve an exhaustive knowledge of the exact lipid profile that characterizes the exosomes as a function of their origin. From this perspective, an emerging approach based on the use of a commercial copper TEM grid as a substrate for the detection of FZD10 protein at surface exosome membranes has been proposed as an easy and versatile method for the investigation of biomarker localization on the surface of exosomes in specific gastrointestinal cancers by using immunogold labeling. Moreover, original preliminary insights on the different lipid contents in membranes of exosomes isolated from healthy (HCEC-1CT) and cancer (Caco-2) cells of the human gastrointestinal tract have indicated that differences in the lipid profile between healthy and cancer cells are suitable for providing useful information for the diagnosis of CRC cancer. Finally, the potential of exosomes as nanocarriers for the delivery of therapeutic agents and treatment of gastrointestinal cancers with reduced side effects with respect to conventional therapies has been addressed. To reach such an ambitious goal, further studies on the nature and structure of exosomes and the engineering of their cargo and membranes are required in order to enable the proper manipulation of exosomes derived from biological fluids for their specific use in anticancer personalized therapies for oncological patients.

## Figures and Tables

**Table 1:** Mean percentage of the fatty acid composition of exosome membranes extracted from HCEC-1CT and Caco-2 cell lines. All data represent the results of three different experiments (mean % ± SD). *p*-value has been determined by a paired Student t test. The bold font is represent the ration between arachidonic acid and eicosapentanoic acid for both cell lines, with standard deviation.

	HCEC-1CT Mean % ± SD	Caco-2 Mean % ± SD	*p*-Value
SFAs (Saturated fatty acids)	90.24 ± 0.18	90.11 ± 0.89	Ns
MUFAs (Monounsaturated fatty acids)	7.03 ± 0.27	6.83 ± 0.22	Ns
PUFAs (Polyunsaturated fatty acids)	2.73 ± 0.18	3.06 ± 0.26	*0.04*
Omega-6 PUFAs	2.25 ± 0.02	2.64 ± 0.09	*0.0001*
Omega-3 PUFAs	0.48 ± 0.02	0.42 ± 0.06	Ns
Linoleic acid (LA) (C18:2n6)	1.95 ± 0.31	2.01 ± 0.44	Ns
γ-linolenic acid (GLA) (C18:3n6)	0.02 ± 0.01	0.16 ± 0.04	*0.001*
Arachidonic acid (AA) (C20:4n6)	0.18 ± 0.02	0.25 ± 0.04	*0.04*
Eicosapentaenoic acid (EPA) (C20:5n3)	0.26 ± 0.04	0.05 ± 0.03	*0.001*
α-linolenic acid (ALA) (C18: 3n3)	0.22 ± 0.09	0.17 ± 0.04	Ns
Omega-6/omega-3 ratio	4.68 ± 0.36	6.28 ± 0.22	*0.001*
**AA/EPA ratio**	**0.69 ** **±** ** 0.04**	**5.0 ** **±** ** 0.49**	***0.0001***
